# Metabolomic Profile of Aqueous Extracts of Commercial Samples of St John’s Wort (*Hypericum perforatum*) Determined by ^1^H NMR

**DOI:** 10.3390/ijms27156757

**Published:** 2026-07-28

**Authors:** Erick Alejandro Herrera-Jurado, Estefanía de Jesús Terán-Sánchez, Elvia Becerra-Martínez, Luis Gerardo Zepeda-Vallejo

**Affiliations:** 1 Departamento de Química Orgánica, Escuela Nacional de Ciencias Biológicas, Instituto Politécnico Nacional, Prolongación de Carpio y Plan de Ayala s/n, Col. Santo Tomas, Alcaldía Miguel Hidalgo, Mexico City 11340, Mexico; erick.cqb@gmail.com (E.A.H.-J.); estefaniateran92@gmail.com (E.d.J.T.-S.); 2 Centro de Nanociencias y Micro y Nanotecnologías, Instituto Politécnico Nacional, Av. Luis Enrique Erro S/N, Unidad Profesional Adolfo López Mateos, Zacatenco, Delegación Gustavo A. Madero, Mexico City 07738, Mexico

**Keywords:** *Hypericum* spp., St John’s wort, ^1^H NMR, metabolomic profiling, multivariate analysis

## Abstract

This study aimed to characterize the metabolomic profile of aqueous extracts of *Hypericum perforatum* (St. John’s wort) commercial samples using ^1^H NMR spectroscopy, in order to evaluate their chemical composition as consumed in infusion form. Seven retail samples were extracted with water under conditions simulating tea preparation and analyzed using ^1^H NMR. Metabolite identification and quantification were performed with Chenomx^®^ software, followed by multivariate statistical analyses including PCA and OPLS-DA. A total of 37 metabolites were identified, predominantly primary metabolites such as sugars (glucose, fructose), amino acids (glycine, asparagine), short-chain organic acids (malic, citric, and acetic acids), secondary metabolites (trigonelline, chlorogenic acid) and metabolism products (methanol, ethanol, acetic acid and acetone). Notably, key bioactive compounds traditionally associated with *H. perforatum*, such as hypericin and hyperforin, were not detected under the experimental conditions employed. Multivariate analyses revealed significant variability among samples, suggesting differences in composition likely related to origin, processing, or potential adulteration. These findings demonstrate that aqueous preparations of St. John’s wort differ substantially from extracts used in clinical trials, being dominated by primary metabolism rather than specialized bioactive compounds. This has important implications for the interpretation of its therapeutic effects when consumed as tea. Furthermore, the study highlights the utility of NMR-based metabolomics as a robust tool for quality assessment and authentication of herbal products.

## 1. Introduction

The genus *Hypericum* comprises approximately 508 species found in various regions around the world and is widely known. The most extensively studied species in this genus is *Hypericum perforatum*, more commonly known as St John’s wort (SJW) [1].

**Figure 1:**
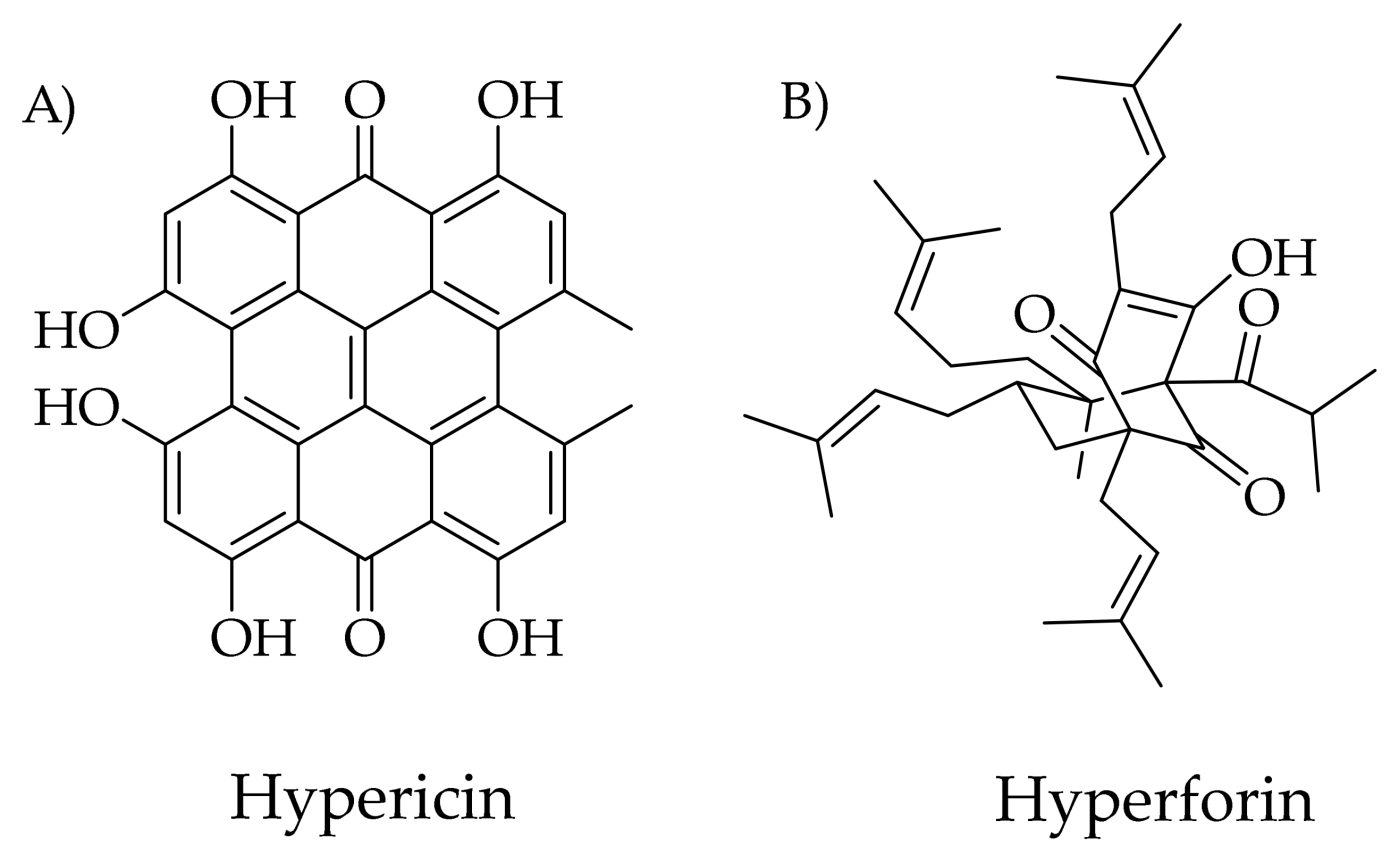
Structure of (**A**) Hypericin and (**B**) hyperforin.

Traditionally, *H. perforatum* has been used to treat abdominal pain, superficial wounds and various bacterial infections [2]. This plant is renowned for its medicinal properties, with numerous studies focusing on identifying the molecules responsible for its biological activity. The major chemical classes present in *H. perforatum* include naphthodianthrones, phloroglucinol derivatives, flavonoids, proanthocyanidins, and chlorogenic acid [3]. The two most extensively studied molecules in this species are hypericin and hyperforin (see Figure 1).

Hypericin has demonstrated antiviral activity against various viruses, including HIV. Additionally, its use in photodynamic therapy for cancer treatment has been studied due to its selective activation in tumor cells when exposed to light. Treatment with hypericin has also been observed to improve memory and learning deficits induced by the amyloid-β (Aβ) peptide in Alzheimer’s disease models [4,5]. On the other hand, hyperforin is a polyprenylated acylphloroglucinol derivative. While the antidepressant activity of St. John’s wort was initially attributed to naphthodianthrones, particularly hypericin, clinical studies have demonstrated that hyperforin is primarily responsible for this effect [6]. It acts by inhibiting the reuptake of a wide range of neurotransmitters, including serotonin, dopamine, norepinephrine, glutamate, and gamma-aminobutyric acid (GABA) [7]. Despite the many reported biological activities, the actual presence and concentration of these compounds in food sources is still uncertain.

The most common method of consuming medicinal plants is through aqueous preparations for oral administration, typically in the form of infusions or teas [8]. From a chemical standpoint, the solubility of compounds depends on the polarity of the solvent. Since water is polar, it tends to dissolve compounds with similar characteristics [9]. Therefore, it is essential to analyze which compounds are transferred to the aqueous phase when preparing infusions for medicinal purposes.

On the other hand, the lack of regulation for plant-based products necessitates the establishment of quality controls capable of detecting adulteration, as it is a persistent public health problem [10]. Frequent consumption of natural products requires the public to have access to more information in order to avoid the risks associated with adulteration, counterfeiting, and poisoning [11]. Various strategies have been developed to identify these irregularities. These strategies range from DNA barcoding to chromatographic and spectroscopic techniques, including infrared (IR), Raman, mass spectrometry, and nuclear magnetic resonance (NMR) [12].

In recent years, metabolomic analyses have become effective tools for quality control in medicinal plants [13]. The primary techniques employed for this purpose are mass spectrometry (MS) and nuclear magnetic resonance (NMR) [14]. NMR offers several advantages. It is a non-destructive technique that preserves the sample for further analysis. It is highly reproducible, enhancing the statistical robustness of studies. Unlike MS, NMR can provide information about the stereochemistry of metabolites [15].

In light of the above, it is necessary to develop an analytical method to authenticate and evaluate the quality of St. John’s wort products available to consumers. This will help to establish identification protocols and ensure the quality and safety of its consumption.

Due to the high consumption of medicinal plants as a first-line remedy for various ailments, accurately defining their metabolomic profiles is critical. While previous studies have centered on bioactive compounds, they often disregard the overall composition of the ingested material. In this regard, we propose using NMR spectroscopy as a reliable and effective method for analyzing and comparing the metabolomic profiles of *Hypericum perforatum* samples obtained from various retail outlets in Mexico City.

## 2. Results

### Aqueous Extracts—Metabolomic Profile of St. John’s Wort Samples

**Figure 2:**
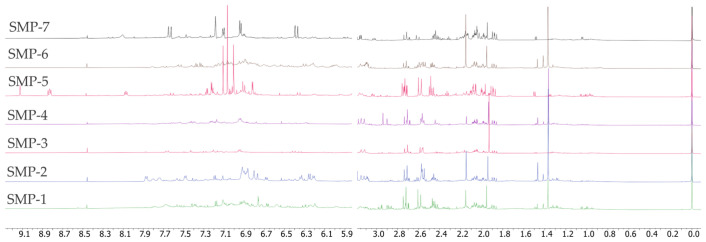
^1^H NMR spectra of samples SMP1–SMP7. High-field (0.00–3.00 ppm) and low-field (5.90–9.10 ppm). SMP = Sample.

One gram of each of the collected samples was weighed and suspended in 10 mL of pre-heated HPLC-grade water; from this, an aliquot was taken to obtain the ^1^H NMR spectra for each sample. The resulting spectra were analyzed using Chenomx^®^ software (v. 12.0). Figure 2 shows the high-field (0.00–3.00 ppm) and low-field (5.90–9.10 ppm) regions for samples SMP1 through SMP7. The most significant differences in the chemical profiles of the analyzed samples are seen at low fields. At this stage, it is important to highlight that variations in a sample’s chemical profile could indicate changes resulting from the collection region, storage conditions, or, in the worst-case scenario, product adulteration.

**Figure 3:**
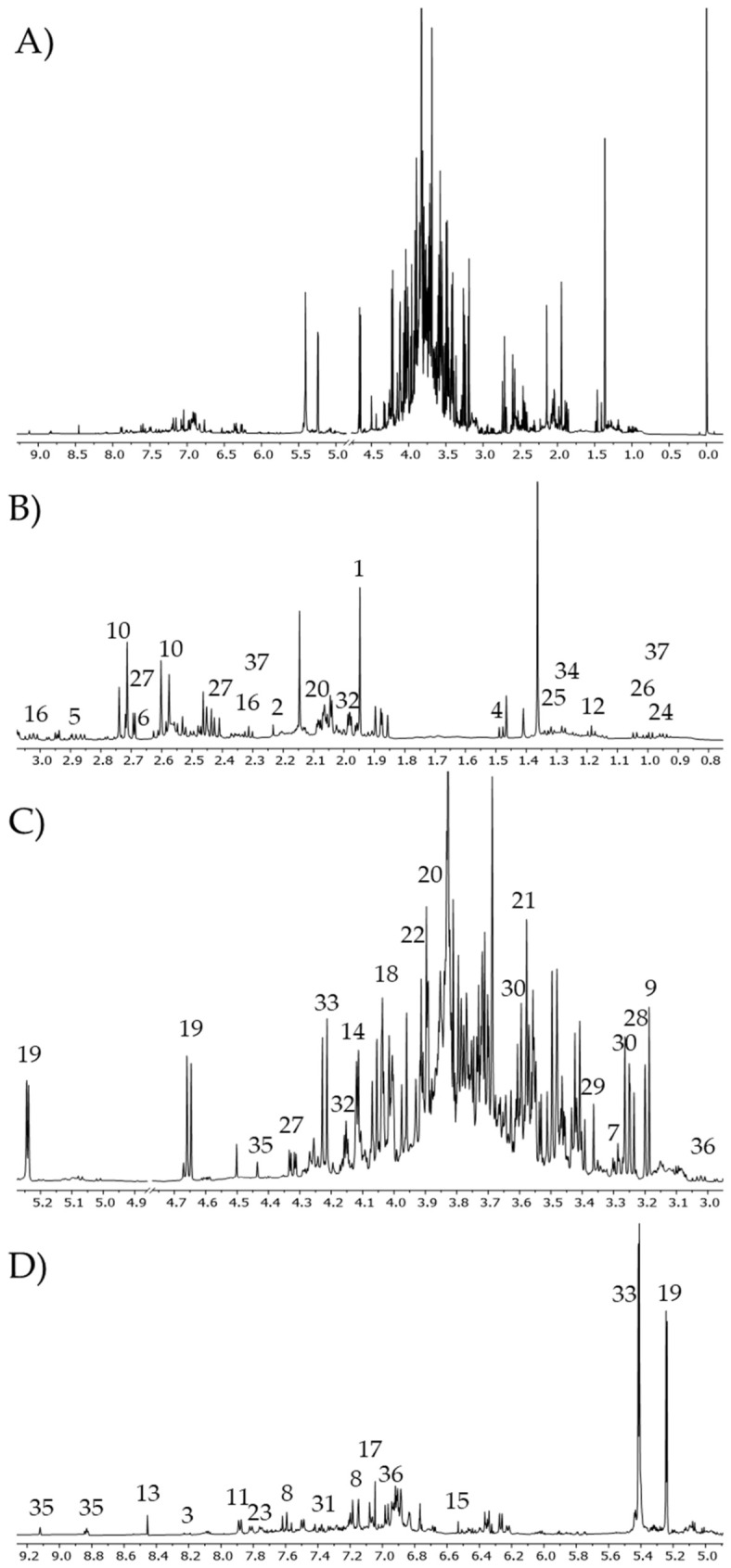
^1^H NMR spectrum (600 MHz) of the aqueous extract of St. John’s Wort shown in several expanded sections: (**A**) δ 0.0–9.2; (**B**) δ 0.8–3.0; (**C**) δ 3.0–5.2; (**D**) δ 5.0–9.2. 1. Acetic acid, 2. Acetone, 3. Adenine, 4. Alanine, 5. Asparagine, 6. Aspartic acid, 7. Betaine, 8. Chlorogenic acid, 9. Choline, 10. Citric acid, 11. Cytidine, 12. Ethanol, 13. Formic acid, 14. Fructose, 15. Fumaric acid, 16. Gamma-aminobutyric acid (GABA), 17. Gallic acid, 18. Glucaric acid, 19. Glucose, 20. Glutamic acid, 21. Glycine, 22. Glycolic acid, 23. Guanosine, 24. Isoleucine, 25. Lactic acid, 26. Leucine, 27. Malic acid, 28. Malonic acid, 29. Methanol, 30. Myo-inositol, 31. Phenylalanine, 32. Proline, 33. Sucrose, 34. Threonine, 35. Trigonelline, 36. Tyrosine, and 37. Valine.

Figure 3 shows the ^1^H NMR spectrum obtained from a pool of SMP1–SMP7. This spectrum reveals the presence of sugar-type molecules, amino acids, and short-chain acids. The identified metabolites are listed in the caption of Figure 3.

**Figure 4:**
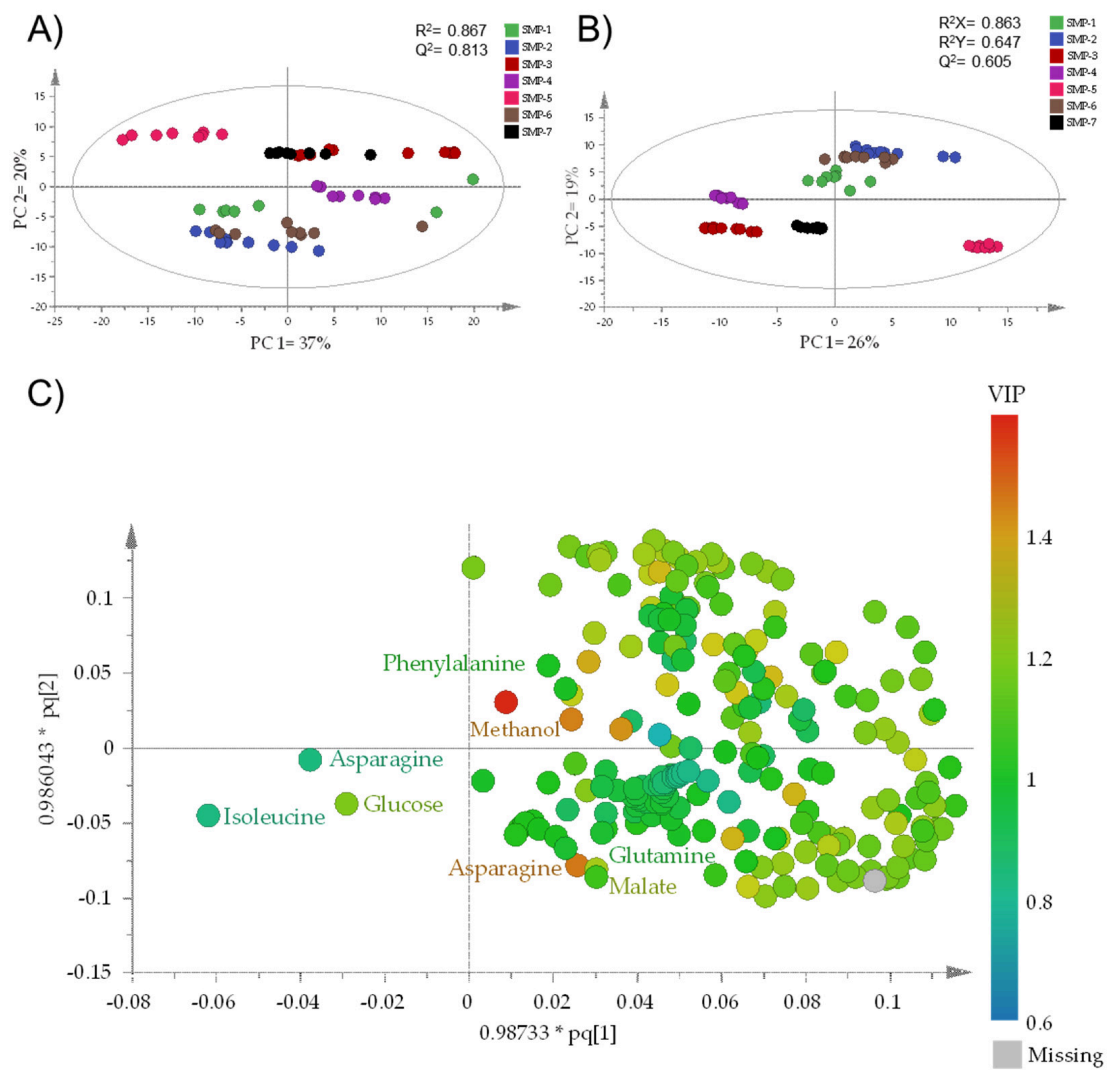
(**A**) PCA, (**B**) OPLS-DA, and (**C**) loading plot of aqueous St. John’s wort samples. 0.9873*pq[1]: Component 1 explaining 98.73% of the variability of the data. 0.9860*pq[2]: Component 2 explaining 98.60% of the variability of the data.

The ^1^H NMR spectra obtained from the aqueous extraction were converted into numerical matrices for statistical analysis. By using the full spectra and a binning approach, a data matrix was generated to reveal similarities in the metabolomic profiles of the samples. First, a Principal Component Analysis (PCA) was performed to observe the natural distribution of the samples. Subsequently, Orthogonal Partial Least Squares (OPLS-DA) was conducted to maximize group separation (Validation in Figure S1). The initial PCA (Figure 4A) yielded R^2^ = 0.867 and Q^2^ = 0.813, with the first two components accounting for 37.0% and 20.0% of the total variance, respectively. Similar values were obtained for the OPLS-DA model (Figure 4B): R^2^X = 0.863, R^2^Y = 0.647, and Q^2^ = 0.605, where PC1 explained 26.0% and PC2 explained 19.0% of the variance.

To identify the metabolites that contribute most significantly to sample discrimination based on their concentration profiles, a loading scatter plot analysis was performed (Figure 4C). It was observed that the metabolites with the highest loadings and thus the greatest influence on the separation of the study groups were isoleucine, glucose, asparagine, methanol, phenylalanine, malate, and glutamine. These findings highlight the primary chemical constituents driving the metabolic variance and the distinct clustering observed between the experimental conditions.

**Figure 5:**
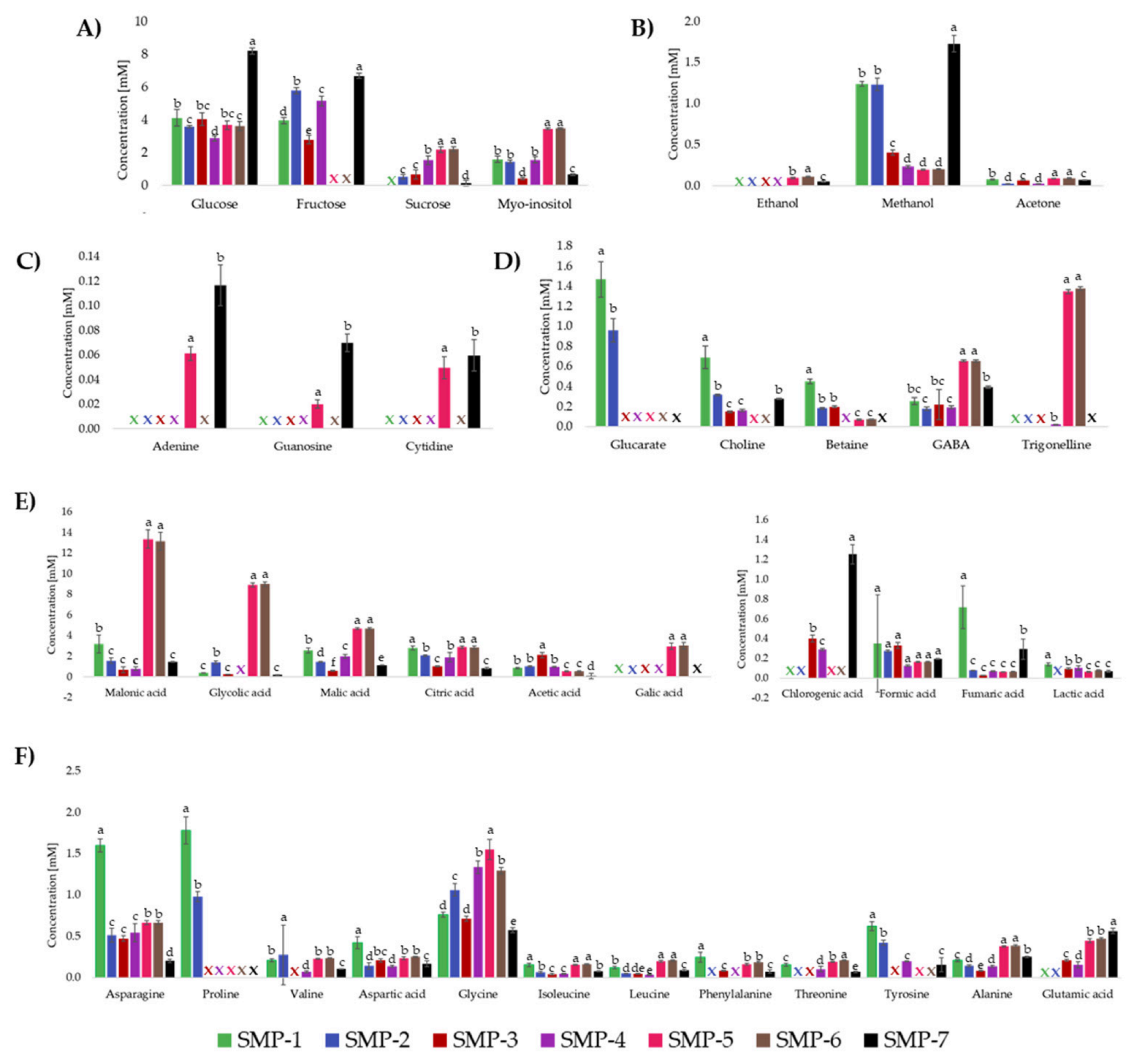
Bar chart of the relative concentrations of the metabolites identified in the metabolome of St. John’s wort tea. (**A**) Sugars and a polyol, (**B**) volatile compounds, (**C**) nitrogenous bases, (**D**) other compounds, (**E**) short-chain organic acids and (**F**) amino acids. The letters above the error bars correspond to the ANOVA analysis by Tukey, performed in InfoStat (v. 2017) with *p* < 0.05. The ‘X’ indicates that a specific metabolite is absent from the sample.

Quantification of the metabolites identified in each sample was carried out for multivariate component statistical analysis and analysis of variance (ANOVA) (Table S1). Figure 5 (see Statistical analysis and validation in Figure S2) shows that glucose and fructose are the predominant sugars, whereas sucrose is present in smaller amounts. Myo-inositol was detected in all samples, with higher concentrations in SMP-5 and SMP-6 and lower concentrations in SMP-3 and SMP-7.

In the volatile metabolite fraction, methanol and acetone were present in all samples. Methanol was the most abundant compound, particularly in SMP-1 and SMP-2. Ethanol was also detected only in SMP-5, SMP-6, and SMP-7.

Nitrogenous bases and nucleosides (adenine, guanosine, and cytidine) were identified exclusively in SMP-4 and SMP-6, with SMP-6 showing the highest concentrations.

Glucarate was observed only in SMP-1 and SMP-2. GABA was detected in all samples, with higher concentrations in SMP-5, SMP-6, and SMP-7. Trigonelline was identified in SMP-5 and SMP-6, and at lower levels in SMP-4. Choline showed its highest concentration in SMP-1, lower concentrations in SMP-2, SMP-3, SMP-4, and SMP-7, and was absent in SMP-5 and SMP-6. Betaine was detected in five samples (SMP-1, SMP-2, SMP-3, SMP-5, and SMP-6), with the highest concentration in SMP-1.

Ten organic acids were identified, of which six (malonic, malic, citric, acetic, formic, and fumaric acids) were present in all samples. Glycolic, gallic, chlorogenic, and lactic acids showed differential distribution. Formic, fumaric, and lactic acids exhibited the lowest concentrations. SMP-5 and SMP-6 showed the highest concentrations of malonic, glycolic, malic, citric, and gallic acids. Notably, gallic acid was detected exclusively in SMP-5 and SMP-6, whereas chlorogenic acid was identified in SMP-3, SMP-4, and SMP-7, with the highest concentration in the latter.

A total of 12 amino acids were identified, of which six (asparagine, aspartic acid, glycine, isoleucine, leucine, and alanine) were present in all samples of St. John’s wort tea. Proline was detected only in SMP-1 and SMP-2. Valine was absent in SMP-3; phenylalanine in SMP-2 and SMP-4; threonine in SMP-2 and SMP-3; and tyrosine in SMP-3, SMP-5, and SMP-6. Glutamic acid was not detected in SMP-1. Overall, proline, valine, phenylalanine, threonine, tyrosine, and glutamic acid showed variability in their presence across samples. Glycine, asparagine, and proline were the most abundant amino acids in the analyzed metabolome.

The ANOVA revealed significant differences (*p* < 0.05) in the concentrations of various metabolites among the commercial samples of St. John’s Wort extract. Sugars, organic acids, and amino acids exhibited the greatest variability, with malic acid, glycolic acid, fructose, and glycine showing the largest differences among samples. Additionally, compounds such as trigonelline and chlorogenic acid displayed notable variations, suggesting differences in phytochemical composition as well as in processing or cultivation conditions among the analyzed brands.

A multivariate analysis was performed using only the relative concentrations of the 37 metabolites identified and quantified using Chenomx^®^ NMR Suite (Chenomx Inc., Edmonton, AB, Canada) in the metabolome of St. John’s wort extracts from different commercial brands.

**Figure 6:**
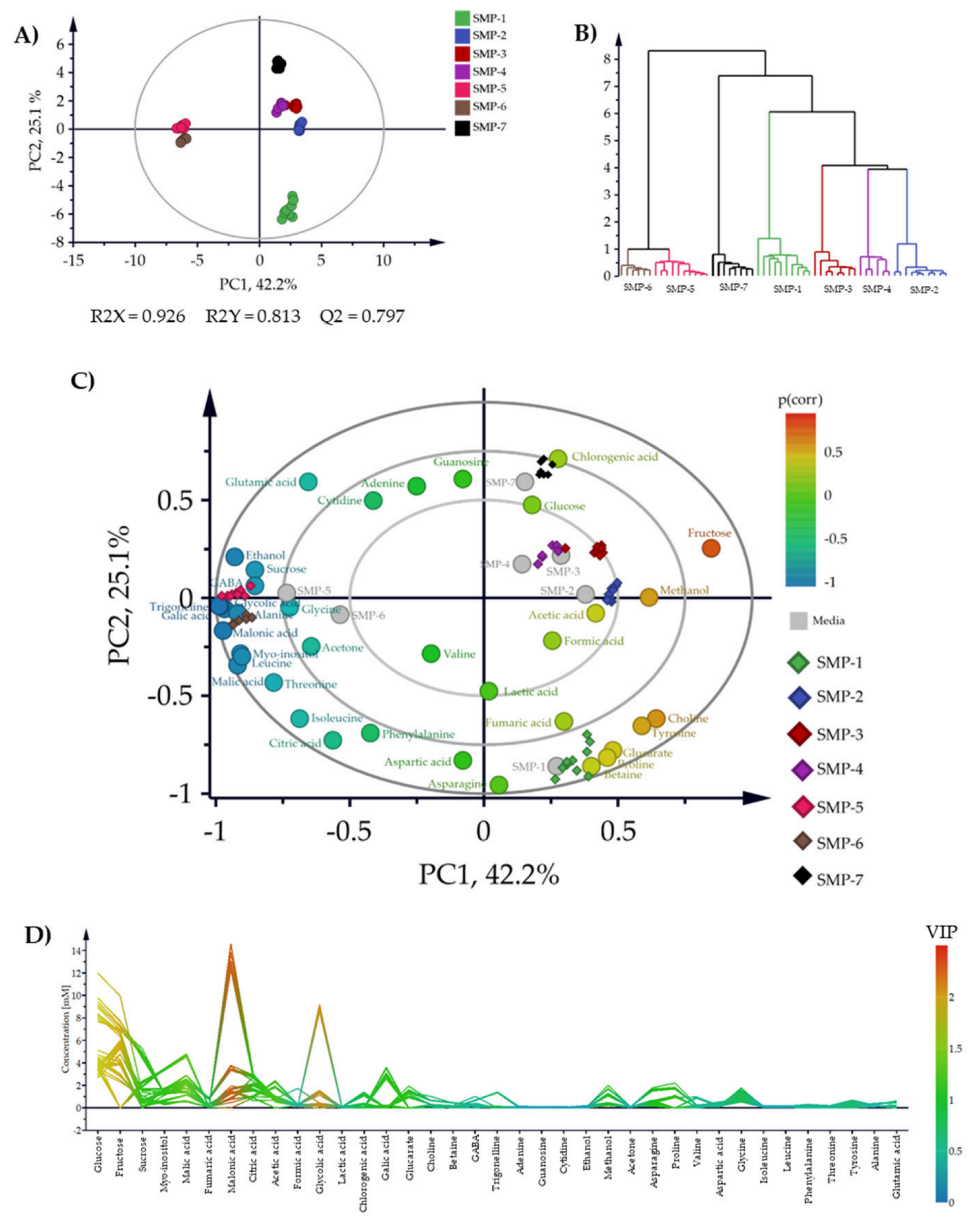
Multivariate analysis of the relative quantification of the metabolites identified and quantified using Chenomx^®^. (**A**) OPLS-DA, (**B**) dendrogram, (**C**) B-Plot, and (**D**) observation spectra plot.

In Figure 6, the OPLS-DA model (Figure 6A) shows the presence of three main groups: one corresponding to SMP-1, a second comprising SMP-5 and SMP-6, and a third including the remaining samples, within which SMP-7 stands out as the most differentiated. This observation was confirmed by the dendrogram (Figure 6B).

The B-plot (Figure 6C) revealed that sample SMP-1 is mainly associated with changes in choline, tyrosine, glucarate, proline, and betaine. In contrast, SMP-2, SMP-3, and SMP-4 show stronger variations in acetic acid, methanol, fructose, and formic acid. Sample SMP-7 exhibits a higher presence of chlorogenic acid, glucose, and guanosine. Meanwhile, SMP-5 and SMP-6 are more strongly associated with changes in trigonelline, gallic acid, malonic acid, alanine, glycolic acid, GABA, sucrose, ethanol, myo-inositol, leucine, and malic acid, making these the samples associated with the highest number of metabolites.

Figure 6D shows that sugars and short-chain organic acids are the most abundant metabolites in the metabolome of St. John’s wort extract. However, malic acid exhibited the greatest variation among samples (VIP ≥ 2), followed by glycolic acid, fructose, and glucose. In turn, gallic acid, myo-inositol, citric acid, acetic acid, glucarate, methanol, asparagine, proline, and glycine showed moderate variation (VIP ≥ 1), while the remaining metabolites exhibited lower variability among samples. These results are consistent with the trends observed in the ANOVA analysis of the quantification matrix.

**Figure 7:**
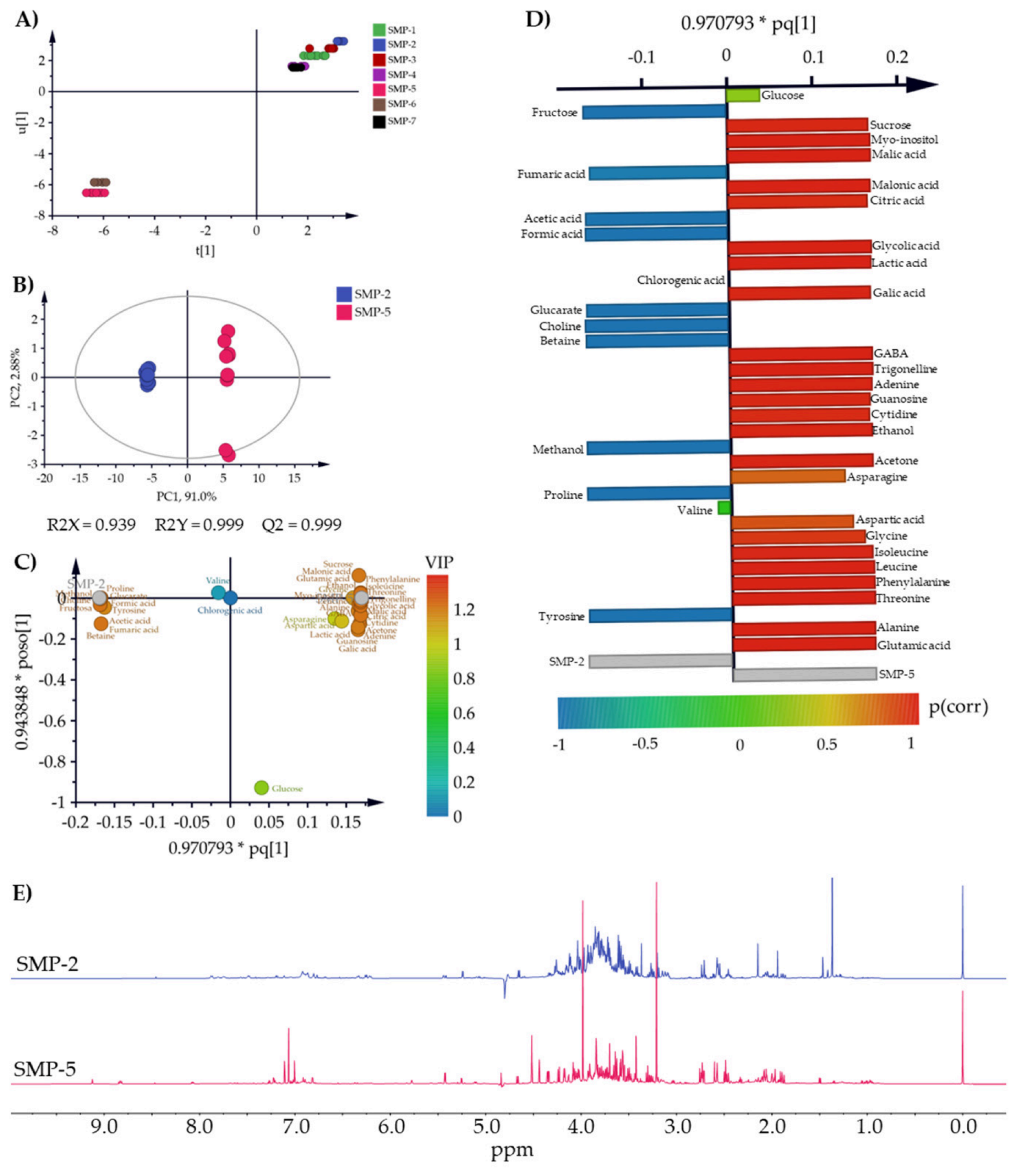
Multivariate analysis of SMP-2 and SMP-5. (**A**) Inner correlation, (**B**) OPLS-DA, (**C**) loading plot, (**D**) p(corr) plot, and (**E**) ^1^H NMR of samples SMP-2 and SMP-5. 0.9707*pq[1]: Component 1 explaining 97.01% of the variability of the data. 0.9438*pq[2]: Component 2 explaining 94.38% of the variability of the data.

An Inner Correlation analysis (Figure 7A), derived from the OPLS-DA model of the multivariate analysis of all commercial samples (Figure 6A), revealed that samples SMP-2 and SMP-5 are the most distinct from each other, enabling their clear discrimination in the OPLS-DA model (Figure 7B) (Validation in Figure S3).

Based on the loading scatter plot (Figure 7C), only glucose, valine, and chlorogenic acid exhibited VIP values ≤ 1, indicating that the remaining metabolites contribute significantly to the differentiation between both samples, reflecting substantial changes in their chemical profiles.

Consistent with these findings, the p(corr) bar plot (Figure 7D) shows predominantly correlations of +1 and −1 between both samples. In particular, SMP-5 is the sample with the highest number of correlations influencing its chemical profile, showing negative correlations with 11 metabolites (fructose, fumaric acid, acetic acid, formic acid, chlorogenic acid, glucarate, choline, betaine, methanol, proline, and valine) and positive correlations with the remaining 26 metabolites. In contrast, these same 11 metabolites show positive correlations with SMP-2, while SMP-2 exhibits negative correlations with the other 26 metabolites identified in the metabolome of St. John’s Wort extract. This information can be explained by analyzing the ^1^H NMR spectra of both samples (Figure 7E), where each spectrum exhibits a distinct and characteristic profile, even though both samples are sold as St. John’s Wort.

## 3. Discussion

The present study uses ^1^H NMR to provide a metabolomic characterization of aqueous extracts of *Hypericum perforatum* commercial samples, revealing a profile dominated by primary metabolites such as sugars, amino acids, and short-chain organic acids. Notably, key bioactive compounds traditionally associated with *H. perforatum*, such as hypericin and hyperforin, were not detected, likely due to concentrations falling below the detection limit of the NMR technique. Wu and Tatsis highlight the limited transfer of these constituents into aqueous preparations [16].

This finding is consistent with previous reports indicating that hypericin and hyperforin exhibit low water solubility and are more efficiently extracted using organic solvents or hydroalcoholic systems [17,18]. Recent studies have demonstrated that infusion-based preparations of *H. perforatum* are chemically distinct from standardized extracts used in clinical settings, which are typically enriched in lipophilic constituents [19]. It is important to note that the aqueous extracts were obtained at 80 °C, which may not be a suitable temperature for extracting the active compounds responsible for St. John’s wort’s therapeutic effects. In fact, it has been demonstrated that the decoction of this plant prepared at the boiling point of water does contain detectable concentrations of flavonoids, hypericin and hyperforin, among other bioactive compounds [17]. This suggests that the pharmacological effect commonly associated with St. John’s wort can be achieved at this temperature.

The predominance of sugars such as glucose and fructose, along with organic acids like malic, citric, and acetic acid, suggests that the aqueous extracts consist mainly of compounds derived from the plant’s primary metabolism. These compounds may contribute to organoleptic properties and mild physiological effects [20], but they are unlikely to be responsible for the well-documented neuropharmacological activity of this plant [21].

Multivariate analyses (PCA, OPLS-DA) revealed significant variability among commercial samples, indicating heterogeneity in their metabolomic composition. This variability may arise from differences in cultivation conditions, plant part used, post-harvest processing, storage, or even species misidentification and adulteration [22]. Similar variability has been reported in recent metabolomic studies of herbal products, emphasizing the need for robust quality control strategies [23].

Interestingly, metabolites such as GABA, trigonelline, and chlorogenic acid were differentially distributed among samples. These compounds have been associated with neuroactive, antioxidant, and metabolic effects, suggesting that aqueous extracts may still possess biological activity, albeit distinct from that of standardized extracts [24,25]. In particular, the presence of GABA across all samples raises questions about its potential contribution to mild anxiolytic effects reported in traditional use [26].

The detection of methanol and acetone in all samples is noteworthy and may be associated with natural plant metabolism, microbial activity, or processing artifacts. However, their consistent presence also underscores the importance of monitoring volatile compounds as part of safety and quality assessments [27].

From a metabolomic perspective, the high reproducibility and non-destructive nature of NMR allowed for robust comparative analysis across samples [28]. The identification of key discriminant metabolites (e.g., malic acid, glycolic acid, fructose, glycine) suggests that these compounds could serve as chemical markers for classification and quality control of *H. perforatum* products.

Overall, this study demonstrates that aqueous preparations of St. John’s wort differ substantially from standardized extracts in terms of chemical composition [29]. These findings have important implications for both consumers and regulatory agencies, as they highlight the need to distinguish between different modes of preparation when evaluating efficacy and safety. In this sense, it is important to note that many commonly consumed herbal products, such as St. John’s wort, lack traceability controls covering planting conditions, growing location, harvesting, distribution, storage conditions and shelf life. Added to this is the lack of species authentication by a botanist or other relevant expert. The uncertainty surrounding these steps prior to a product being made available for human consumption can affect the efficacy of herbal medicines.

On the other hand, the chemical composition of aqueous extracts described here differs from that reported by Alahmad et al. [17], who conducted a comprehensive HPLC-DAD–LC-ESI-QTOF-MS workflow on the composition of aqueous, ethanolic, methanolic and acetone extracts, placing special emphasis on secondary metabolites, particularly flavonoids, hyperforin and hypericin, following a targeted protocol. In contrast, by using NMR as an analytical platform, the present study prioritized the analysis of primary metabolites in an untargeted context. Another notable difference is that Alahmad et al. focused their study on a single St. John’s wort sample, whereas this untargeted study emphasizes the variability between batches of commercial St. John’s wort samples. We believe that the latter approach is ideal for applying authenticity and quality control criteria to this widely consumed medicinal plant. Both studies make an important scientific contribution to our comprehensive understanding of the chemical profiles of St. John’s wort.

Although only commercial samples were considered at this stage of our research, we believe that subsequent studies should include a botanically certified sample to serve as a reference for bio-phytoequivalence or designation of origin studies. The first case must involve developing an extraction protocol that correlates with the best biological activity.

Future studies should integrate NMR-based metabolomics with LC-MS approaches to capture both polar and non-polar metabolites, as well as incorporate DNA barcoding to confirm botanical identity [30]. Additionally, correlating metabolomic profiles with biological activity assays would provide a more comprehensive understanding of the functional properties of these preparations.

## 4. Materials and Methods

### 4.1. Collection and Preparation of Botanical Material

In 2023, seven retail samples (SMP-1 to SMP-7) of herbal remedies were collected from various markets and specialty stores in Mexico City. All samples were mechanically ground into a powder, stored in the dark, and processed.

### 4.2. Preparation of Aqueous Extracts of St. John’s Wort

One gram of each powdered sample (SMP1–SMP-7) was placed in 10 mL of preheated HPLC-grade water (Fermont, Productos Químicos Monterrey, SA de CV, Monterrey, Mexico) at 80 °C and allowed to stand for five minutes. Ten independent replicates were performed for each sample. The resulting extracts were filtered through a 0.22-μm hydrophilic filter. After filtering the samples, 400 μL of each was transferred to an NMR tube. Then, 140 μL of phosphate buffer at pH 5, 60 μL of a 10 mM standard solution of 3-trimethylsilylpropionic acid sodium salt (TSP), and 10 mM EDTA were added to the tube. This resulted in a final concentration of 1 mM TSP and EDTA and a final volume of 600 μL.

### 4.3. ^1^H NMR Spectrum Acquisition

The ^1^H NMR spectra of the aqueous extracts were obtained using a 600 MHz Bruker Avance III spectrometer (14.1 T, BioSpin, Rheinstetten, Germany) equipped with a 5 mm broadband probe (PA BBO 600S3 BBF-H-D-05 Z SP). The NOESYPR1D pulse sequence was used for water suppression. The measurements were taken at a temperature of 25 °C using the following acquisition parameters: Scan number = 256, FID size = 32 K, spectral width = 16.00 ppm, receiver gain = 56.1, acquisition time = 1.7039 s, relaxation time = 5 s. A total of 7 groups, each with 10 replicates, generated a matrix of 70 ^1^H NMR spectra.

### 4.4. Identification of Metabolites

Metabolites were identified by consulting online databases, such as HMDB (https://www.hmdb.ca/; accessed on 24 April 2026) and NP-MRD (https://np-mrd.org/; accessed on 11 May 2026), as well as using the Chenomx^®^ Suite software (v. 12.0). Finally, the ^1^H NMR spectrum was confirmed by acquiring 2D NMR spectra, such as J-resolved, HSQC, HMBC, and COSY (Figure S4a–e).

### 4.5. Processing of ^1^H NMR Spectra

Each FID (free induction decay) was processed using version 12.0.2-20910 of MestreNova software (v. 14). The baseline and phase were corrected, and the internal standard was calibrated to 0.00 ppm. The ^1^H NMR spectrum was integrated and referenced to the TSP signal. Line broadening was performed at 0.3 Hz. A binning size of 0.04 ppm was used, ranging from 0.20 to 10.00 ppm. For aqueous samples, the water suppression region (4.70–5.00 ppm) was discarded before statistical analysis.

### 4.6. Statistical Analysis

First, a principal component analysis (PCA) was performed, followed by a supervised Orthogonal Projections to Latent Structures Discriminant Analysis (OPLS-DA). Model quality was assessed using the R^2^X, Q^2^ values. All data were normalized using UV scaling. The variable importance projection (VIP > 1) was considered to measure the contribution of the variables that allowed discrimination between groups. These analyses were performed using SIMCA^®^ version 14.1 (Umetrics, Kinnelon, NJ, USA).

To assess the robustness and statistical validity of the developed models, a permutation validation analysis was conducted across 200 iterations and CV-ANOVA (Figures S2 and S3). This procedure was carried out to guarantee that the models’ predictive performance did not occur by chance and to substantiate the statistical significance of the findings.

## 5. Conclusions

This study provides a comprehensive metabolomic characterization of aqueous extracts of *Hypericum perforatum* commercial samples using ^1^H NMR spectroscopy. The results demonstrate that the metabolome of St. John’s wort infusions is predominantly composed of primary metabolites (including sugars, amino acids, and short-chain organic acids), secondary metabolites such as trigonelline and chlorogenic acid, and metabolism products such as methanol and ethanol, with glucose, fructose, malic acid, and glycine being among the most abundant and variable compounds. The absence of key bioactive constituents such as hypericin and hyperforin confirms that these compounds are not effectively extracted under aqueous conditions, reinforcing the notion that herbal tea-based preparations of St. John’s wort differ significantly from standardized extracts commonly used in clinical contexts. Consequently, the pharmacological effects attributed to St. John’s wort may not be directly due to its consumption as an herbal tea.

The observed variability among commercial samples, supported by multivariate and ANOVA analyses, highlights substantial heterogeneity in their chemical composition. This variability may be associated with differences in cultivation, processing or storage conditions, or potential adulteration. This emphasizes the need for stricter quality control when commercializing this plant for human consumption. Future studies integrating complementary analytical techniques and biological assays will be essential to further elucidate the functional implications of these findings. Finally, the presence of volatile compounds, primarily methanol and acetone, in most of the studied samples should be considered a quality control criterion for St. John’s wort intake. Overall, this work underscores the relevance of NMR-based metabolomics as a reliable and reproducible approach for determining the chemical profile and authentication of medicinal plants.

## Data Availability

The original contributions presented in this study are included in the article/Supplementary Materials. Further inquiries can be directed to the corresponding authors.

## Supplementary Materials

The following supporting information can be downloaded at: https://www.mdpi.com/article/10.3390/ijms27156757/s1.

## Abbreviations

The following abbreviations are used in this manuscript:

^1^H NMR	Proton Nuclear Magnetic Resonance
PCA	Principal Component Analysis
OPLS-DA	Orthogonal Partial Least Squares Discriminant Analysis
MS	Mass Spectrometry
IR	Infrared Spectroscopy
HPLC	High-Performance Liquid Chromatography
FID	Free Induction Decay
TSP	3-trimethylsilylpropionic acid sodium salt
EDTA	Ethylenediaminetetraacetic acid
ANOVA	Analysis of Variance
VIP	Variable Importance in Projection
NOESYPR1D	1D Nuclear Overhauser Effect Spectroscopy with Water Presaturation
SJW	St. John’s Wort (*Hypericum perforatum*)
GABA	Gamma-aminobutyric acid
Aβ	Amyloid-beta peptide
HIV	Human Immunodeficiency Virus
R^2^X	Fraction of the variation in the X variables explained by the model
R^2^Y	Fraction of the variation in the Y variables explained by the model
Q^2^	Predictive ability of the model
PC	Principal Component
p(corr)	Correlation coefficient in the loading plot
