# Supplementary material for: Metabolomic Profile of Aqueous Extracts of Commercial Samples of St John’s Wort (Hypericum perforatum) Determined by 1H NMR
Source: Int J Mol Sci. 2026 Jul 28;27(15):6757. doi: 10.3390/ijms27156757 (PMC13467255; doi:10.3390/ijms27156757)

Supplementary Materials

Metabolomic Profile of Aqueous Extracts of Commercial Samples of St John's Wort (*Hypericum perforatum*) Determined by ^{1}H NMR

Erick Alejandro Herrera-Jurado [1], Estefanía de Jesús Terán-Sánchez [1], Elvia Becerra-Martínez [2,*] and Luis Gerardo Zepeda-Vallejo [1,*]

1 Departamento de Química Orgánica, Escuela Nacional de Ciencias Biológicas, Instituto Politécnico Nacional, Prolongación de Carpio y Plan de Ayala s/n, Col. Santo Tomas, Alcaldía Miguel Hidalgo, Mexico City 11340, Mexico; erick.cqb@gmail.com (E.A.H.-J.); estefaniateran92@gmail.com (E.d.J.T.-S.)

2 Centro de Nanociencias y Micro y Nanotecnologías, Instituto Politécnico Nacional, Av. Luis Enrique Erro S/N, Unidad Profesional Adolfo López Mateos, Zacatenco, Delegación Gustavo A. Madero, Ciudad de México 07738, Mexico

* Correspondence: elmartinezb@ipn.mx (E.B.-M.); lzepeda@ipn.mx (L.G.Z.-V.)

Table S1. Relative concentrations (mM) of the metabolites identified in the chemical profile of St. John's wort infusion. NA = Not Available.

	Metabolite	SMP-1	SMP-2	SMP-3	SMP-4	SMP-5	SMP-6	SMP-7	QS
Sugars	Glucose	4.123 ± 0.51	3.579 ± 0.06	4.033 ± 0.39	2.874 ± 0.16	3.671 ± 0.27	3.608 ± 0.27	8.201 ± 0.18	9.593 ± 1.12
	Fructose	3.952 ± 0.18	5.776 ± 0.21	2.785 ± 0.25	5.151 ± 0.30	NA	NA	6.681 ± 0.15	7.682 ± 0.91
	Myo-inositol	1.593 ± 0.19	1.447 ± 0.09	0.428 ± 0.07	1.551 ± 0.19	3.453 ± 0.06	3.462 ± 0.06	0.664 ± 0.05	1.221 ± 0.04
	Sucrose	NA	0.519 ± 0.13	0.656 ± 0.28	1.528 ± 0.27	2.175 ± 0.17	2.210 ± 0.14	0.140 ± 0.17	4.409 ± 1.15
Organic acids	Malonic acid	3.183 ± 0.87	1.568 ± 0.27	0.676 ± 0.27	0.797 ± 0.18	13.348 ± 0.90	13.142 ± 0.88	1.457 ± 0.04	1.387 ± 0.21
	Glycolic acid	0.382 ± 0.02	1.416 ± 0.14	0.247 ± 0.01	NA	8.921 ± 0.20	8.985 ± 0.19	0.179 ± 0.03	1.358 ± 0.04
	Malic acid	2.561 ± 0.23	1.447 ± 0.07	0.596 ± 0.05	1.989 ± 0.19	4.666 ± 0.09	4.659 ± 0.10	1.134 ± 0.03	2.237 ± 0.28
	Citric acid	2.805 ± 0.18	2.063 ± 0.07	1.016 ± 0.05	1.892 ± 0.45	2.885 ± 0.013	2.844 ± 0.14	0.839 ± 0.08	1.703 ± 0.12
	Acetic acid	0.853 ± 0.07	1.021 ± 0.03	2.109 ± 0.25	0.988 ± 0.05	0.556 ± 0.05	0.552 ± 0.05	0.089 ± 0.25	0.843 ± 0.02
	Galic acid	NA	NA	NA	NA	2.966 ± 0.32	3.025 ± 0.31	NA	0.183 ± 0.03
	Chlorogenic acid	NA	NA	0.400 ± 0.03	0.290 ± 0.01	NA	NA	1.252 ± 0.10	0.080 ± 0.02
	Formic acid	0.348 ± 0.49	0.276 ± 0.01	0.330 ± 0.03	0.126 ± 0.01	0.165 ± 0.01	0.166 ± 0.004	0.196 ± 0.01	0.216 ± 0.01
	Fumaric acid	0.717 ± 0.22	0.079 ± 0.001	0.027 ± 0.001	0.068 ± 0.004	0.065 ± 0.002	0.066 ± 0.001	0.294 ± 0.10	0.061 ± 0.004
	Lactic acid	0.141 ± 0.01	NA	0.096 ± 0.01	0.102 ± 0.02	0.065 ± 0.01	0.078 ± 0.01	0.067 ± 0.01	0.067 ± 0.003
Others	Glucarate	1.467 ± 0.18	0.958 ± 0.11	NA	NA	NA	NA	NA	NA
	Choline	0.689 ± 0.11	0.314 ± 0.01	0.147 ± 0.01	0.161 ± 0.01	NA	NA	0.276 ± 0.01	0.253 ± 0.01
	Betaine	0.452 ± 0.02	0.182 ± 0.01	0.196 ± 0.01	NA	0.067 ± 0.01	0.071 ± 0.004	NA	0.010 ± 0.03
	GABA	0.254 ± 0.04	0.178 ± 0.02	0.217 ± 0.15	0.192 ± 0.02	0.654 ± 0.01	0.654 ± 0.01	0.391 ± 0.01	0.023 ± 0.07
	Trigonelline	NA	NA	NA	0.020 ± 0.002	1.345 ± 0.02	1.378 ± 0.02	NA	0.142 ± 0.01
Nucleotides	Adenine	NA	NA	NA	NA	0.061 ± 0.006	NA	0.116 ± 0.017	0.021 ± 0.006
	Guanosine	NA	NA	NA	NA	0.020 ± 0.003	NA	0.070 ± 0.007	0.007 ± 0.013
	Cytidine	NA	NA	NA	NA	0.050 ± 0.009	NA	0.060 ± 0.013	0.028 ± 0.001
Volatile	Ethanol	NA	NA	NA	NA	0.098 ± 0.010	0.112 ± 0.007	0.057 ± 0.007	0.169 ± 0.006
	Methanol	1.242 ± 0.028	1.233 ± 0.075	0.409 ± 0.032	0.235 ± 0.015	0.195 ± 0.007	0.202 ± 0.005	1.730 ± 0.10	0.437 ± 0.013
	Acetone	0.080 ± 0.005	0.025 ± 0.03	0.069 ± 0.004	0.026 ± 0.001	0.090 ± 0.005	0.093 ± 0.004	0.071 ± 0.002	0.051 ± 0.001
Amino acids	Asparagine	1.600 ± 0.08	0.514 ± 0.08	0.474 ± 0.03	0.545 ± 0.11	0.666 ± 0.02	0.669 ± 0.02	0.202 ± 0.02	0.350 ± 0.04
	Proline	1.784 ± 0.17	0.981 ± 0.06	NA	NA	NA	NA	NA	NA
	Valine	0.213 ± 0.02	0.275 ± 0.36	NA	0.071 ± 0.01	0.230 ± 0.01	0.236 ± 0.003	0.105 ± 0.004	0.842 ± 0.29
	Aspartic acid	0.423 ± 0.08	0.141 ± 0.04	0.210 ± 0.02	0.138 ± 0.01	0.235 ± 0.02	0.252 ± 0.01	0.167 ± 0.04	0.094 ± 0.01
	Glycine	0.762 ± 0.03	1.063 ± 0.07	0.716 ± 0.03	1.338 ± 0.08	1.553 ± 0.12	1.295 ± 0.04	0.575 ± 0.03	1.438 ± 0.13
	Isoleucine	0.153 ± 0.02	0.064 ± 0.01	0.044 ± 0.01	0.044 ± 0.01	0.156 ± 0.01	0.163 ± 0.004	0.078 ± 0.01	0.055 ± 0.002
	Leucine	0.122 ± 0.02	0.050 ± 0.003	0.045 ± 0.01	0.035 ± 0.003	0.198 ± 0.01	0.208 ± 0.01	0.091 ± 0.01	0.054 ± 0.003
	Phenylalanine	0.247 ± 0.06	NA	0.081 ± 0.01	NA	0.162 ± 0.01	0.188 ± 0.01	0.073 ± 0.02	0.072 ± 0.003
	Threonine	0.155 ± 0.02	NA	NA	0.104 ± 0.03	0.192 ± 0.01	0.213 ± 0.01	0.068 ± 0.01	0.080 ± 0.003
	Tyrosine	0.624 ± 0.05	0.422 ± 0.03	NA	0.201 ± 0.01	NA	NA	0.158 ± 0.09	0.292 ± 0.01
	Alanine	0.208 ± 0.01	0.145 ± 0.01	0.082 ± 0.005	0.138 ± 0.009	0.378 ± 0.09	0.390 ± 0.01	0.252 ± 0.01	0.132 ± 0.006
	Glutamic acid	NA	NA	0.209 ± 0.01	0.153 ± 0.03	0.448 ± 0.03	0.473 ± 0.01	0.565 ± 0.03	0.199 ± 0.5

Figure S1. OPLS-DA Validation of the binning matrix for St. Jhon's wort aqueous extracts

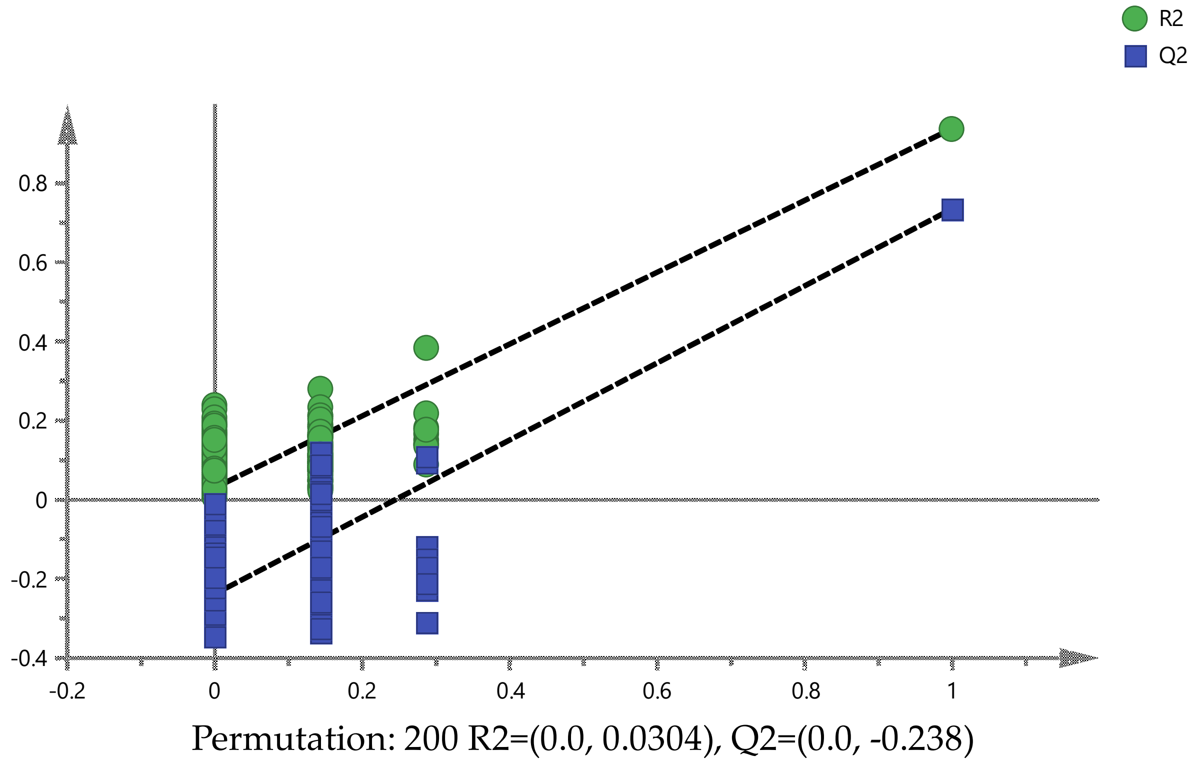

The supervised model was further validated using a 200-permutation test. The original model exhibited high goodness-of-fit ($R^2 \approx 0.92$) and predictive ability ($Q^2 \approx 0.73$), whereas the permuted models showed substantially lower R^2 and Q^2 values. The regression line intercepts were 0.0304 for R^2 and −0.238 for Q^2. The negative Q^2 intercept indicates that the observed class discrimination was not due to random chance and confirms the absence of model overfitting. Collectively, these results demonstrate the robustness, reliability, and predictive validity of the supervised model.

Figure S2. Statistical analysis and validation of the full set of sample analysis. A) PCA, B) PLS-DA, C) Permutation analysis and D) Cross Validation by ANOVA.

A)

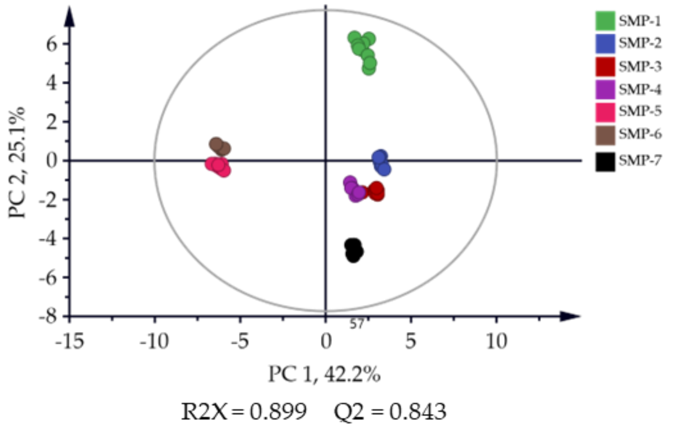

B)

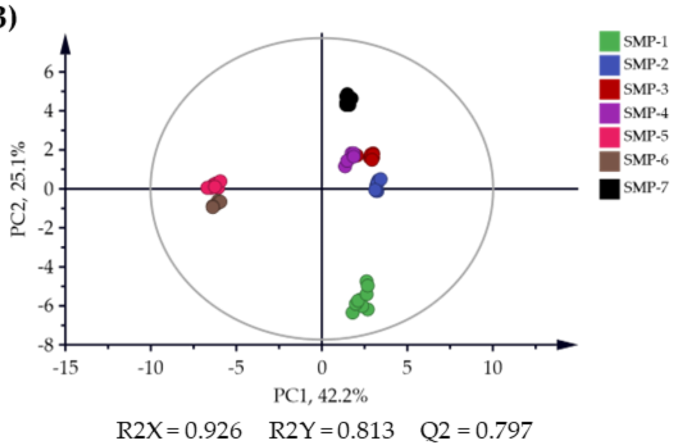

C)

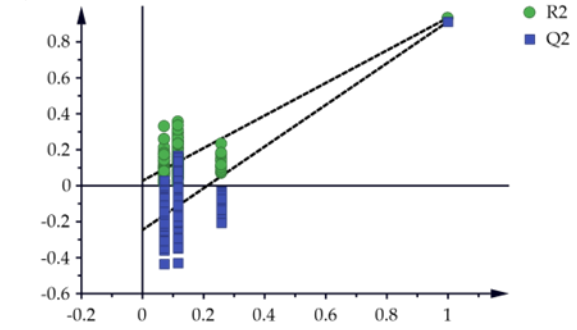

D)

CV-ANOVA	SS	DF	MS	F	p	SD
**Total corr.**	342	342	1			1
Regression	262.336	60	4.372	15.477	0	2.091
**Residual**	79.664	282	0.282			0.531

The statistical significance of the supervised model was evaluated using cross-validated analysis of variance (CV-ANOVA). The model showed an F-value of 15.477 and a p-value < 0.001, indicating that the variation explained by the model was significantly greater than the residual variation. In addition, the regression sum of squares (SS = 262.336) accounted for approximately 76.7% of the total variation, whereas the residual sum of squares (SS = 79.664) represented 23.3%, demonstrating the robustness of the model. Together with the R^2Y and Q^2 values and the permutation test, these results confirm the robustness and predictive reliability of the model for identifying the metabolites responsible for discriminating among the different samples.

Figure S3. Statistical analysis and validation of samples SMP-2 and SMP-5. A) PCA, B) PLS-DA, C) Permutation analysis and D) Cross-validation by ANOVA.

A)

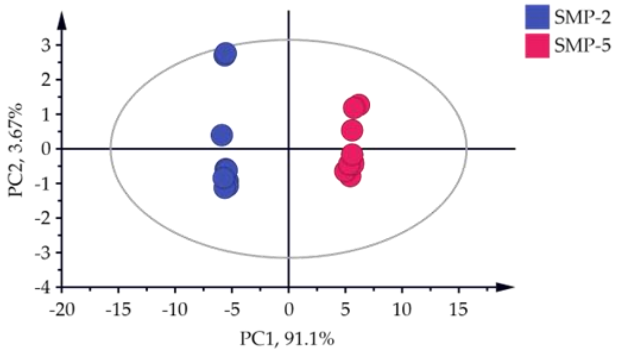

R2X = 0.948 Q2 = 0.891

B)

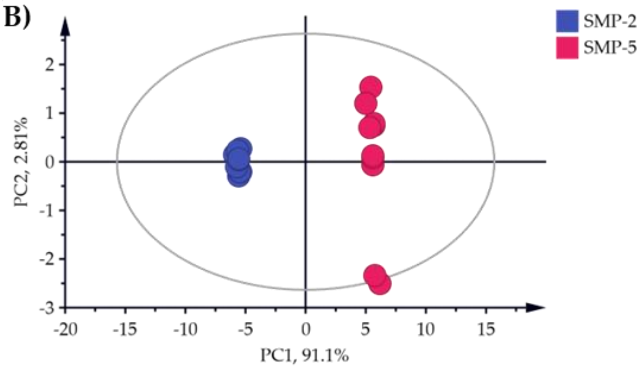

R2X = 0.939 R2Y = 0.999 Q2 = 0.999

C)

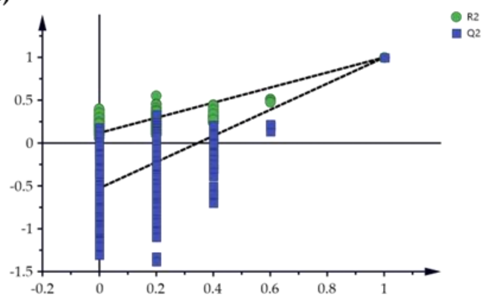

D)

CV-ANOVA	SS	DF	MS	F	p	SD
Total corr.	19	19	1			1
Regression	18.972	4	4.743	2544.65	$4.864x10^{-21}$	2.178
Residual	0.028	15	0.001			0.043

The statistical significance of the supervised model was evaluated using cross-validated analysis of variance (CV-ANOVA). The model showed an F-value of 2544.65 and a p-value < 0.001, indicating that the variation explained by the model was significantly greater than the residual variation. In addition, the regression sum of squares (SS = 262.336) accounted for approximately 99.85% of the total variation, whereas the residual sum of squares (SS = 0.028) represented 0.15%, demonstrating the robustness of the model. Together with the R^2Y and Q^2 values and the permutation test, these results confirm the robustness and predictive reliability of the model for identifying the metabolites responsible for discriminating among the different samples.

Figure S4a. ^{1}H NMR spectrum of the aqueous extract of St. John's wort (*Hypericum perforatum),* used for the confirmation of its metabolomic profile.

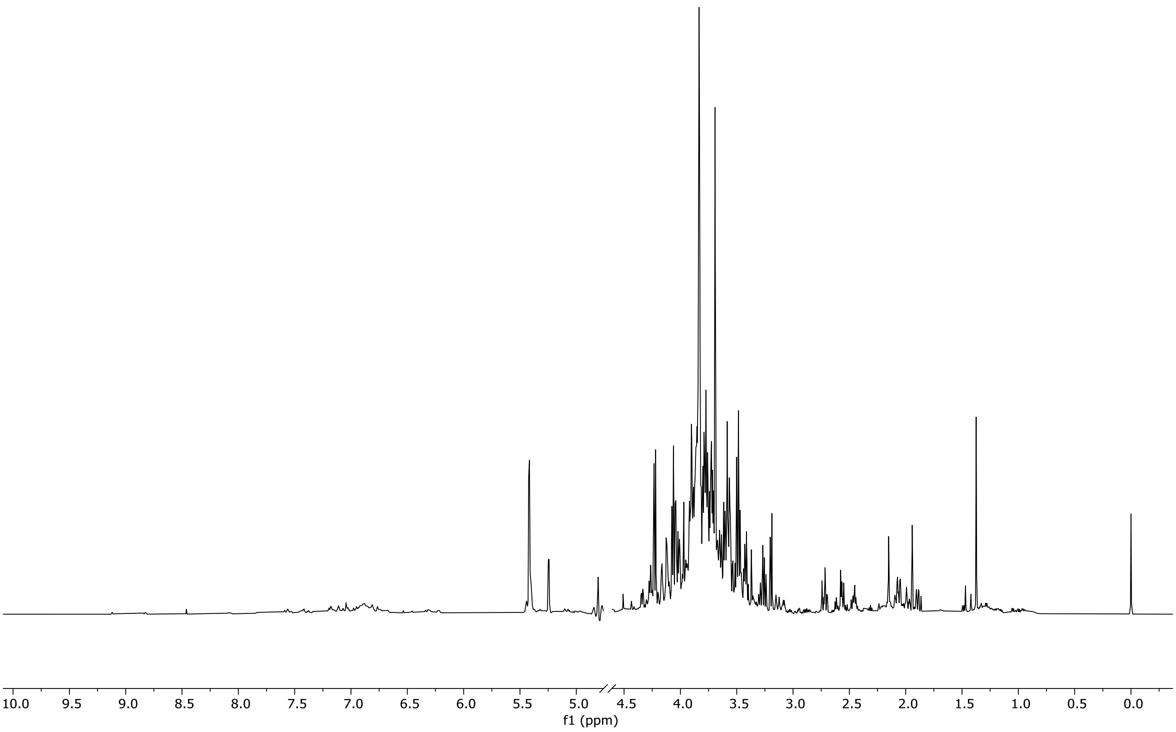

Figure S4b. HSQC NMR spectrum of the aqueous extract of St. John's wort (*Hypericum perforatum*), used to identify the main metabolites present in its metabolomic profile.

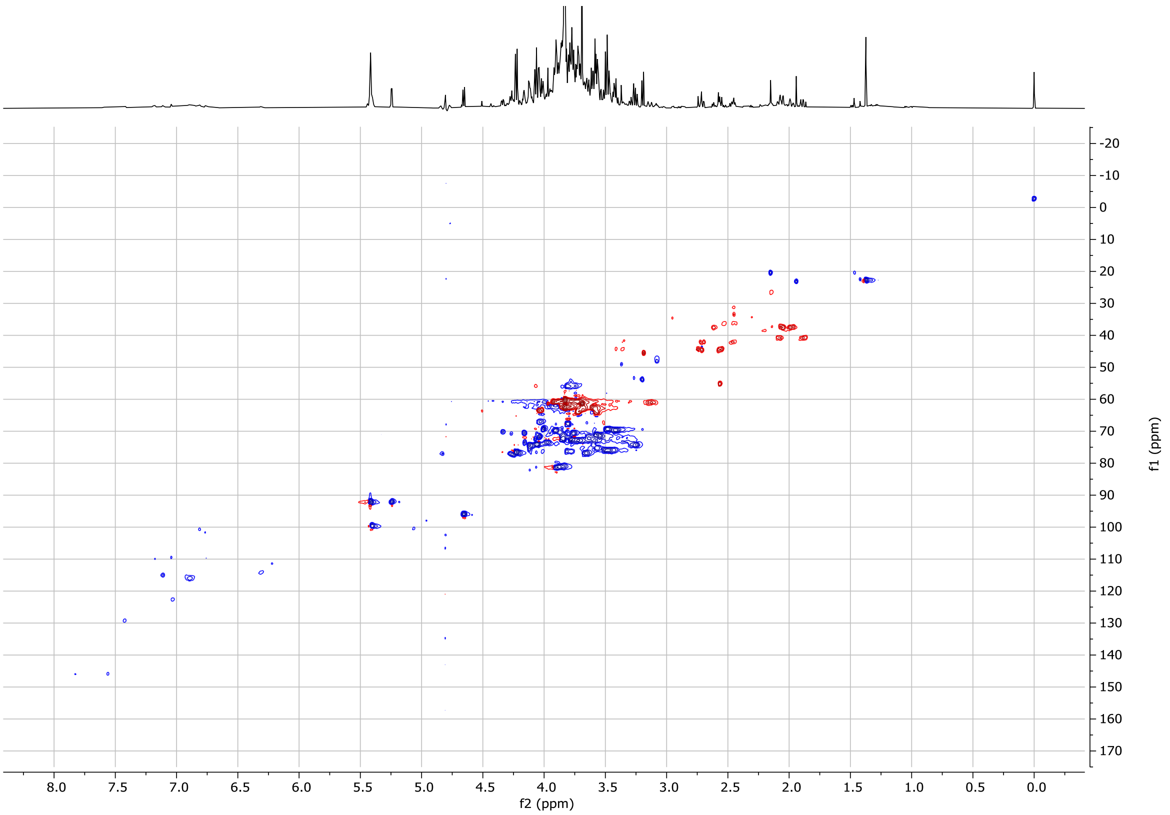

Figure S4c. HMBC NMR spectrum of the aqueous extract of St. John's wort (*Hypericum perforatum*), used to identify the main metabolites present in its metabolomic profile.

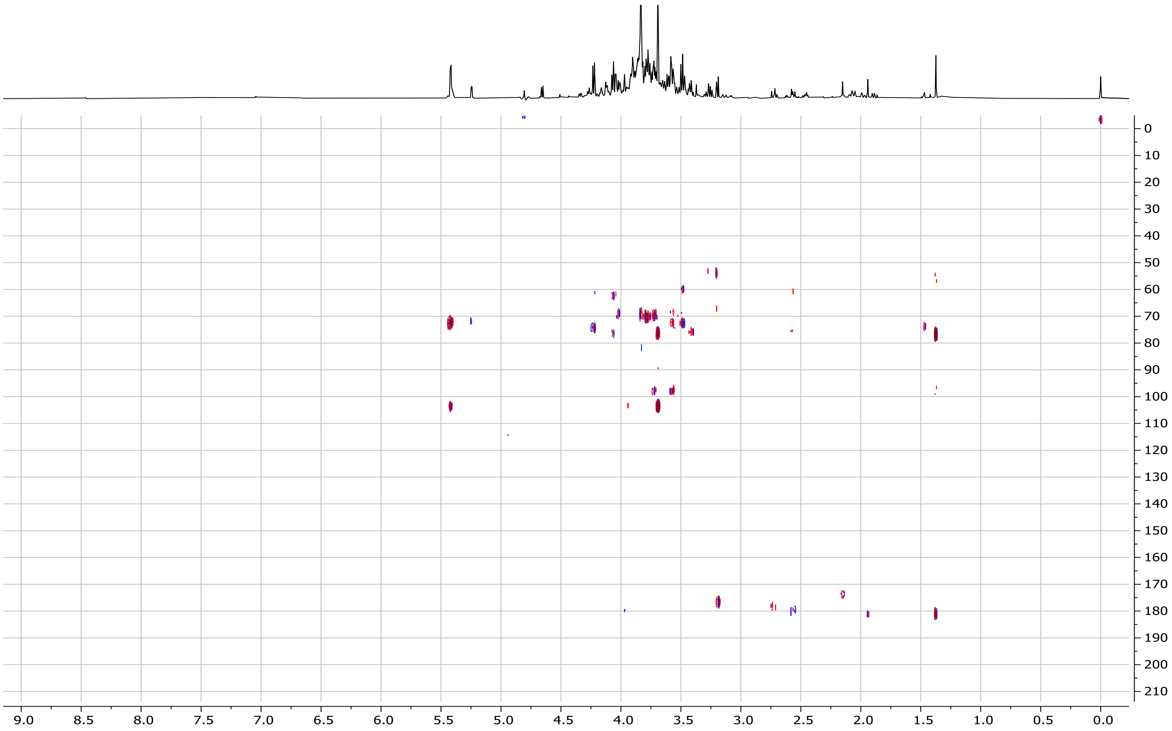

Figure S4d. COSY NMR spectrum of the aqueous extract of St. John's wort (*Hypericum perforatum*), used to identify the main metabolites present in its metabolomic profile.

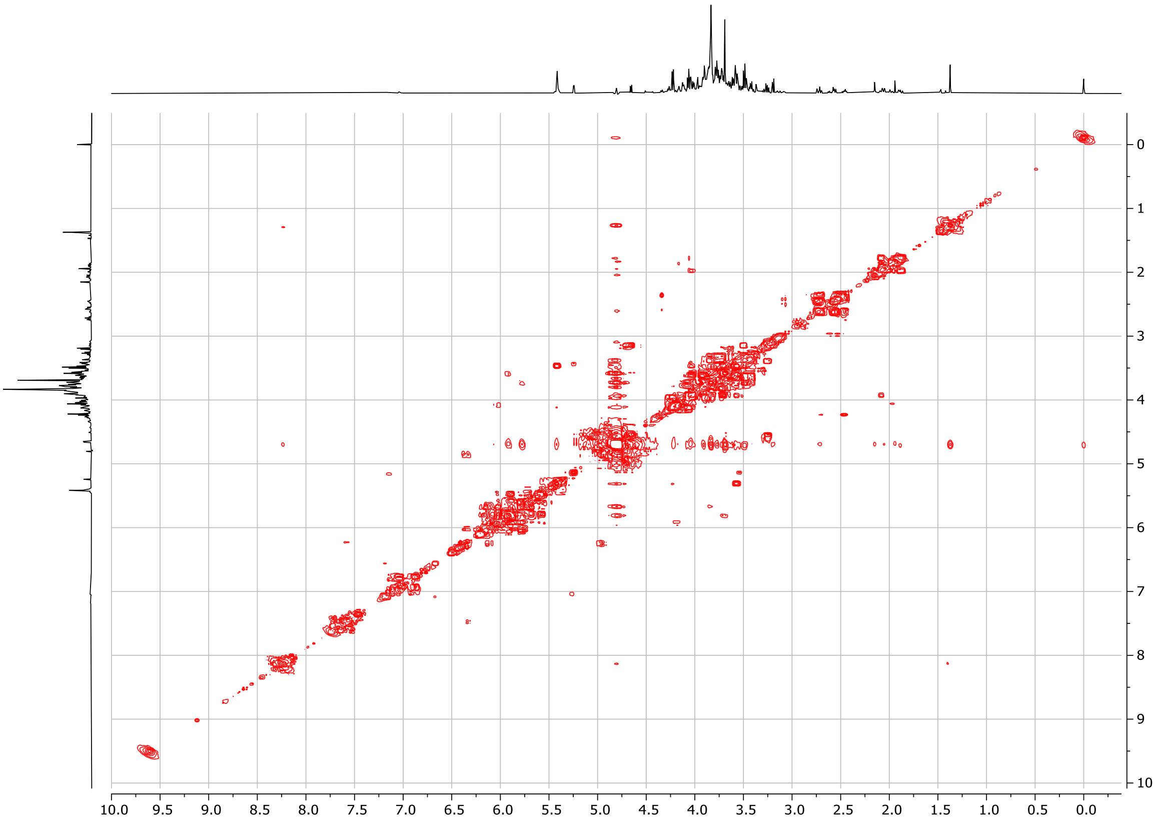

Figure S4e. *J*-resolved NMR spectrum of the aqueous extract of St. John's wort (*Hypericum perforatum*), used to identify the main metabolites present in its metabolomic profile.

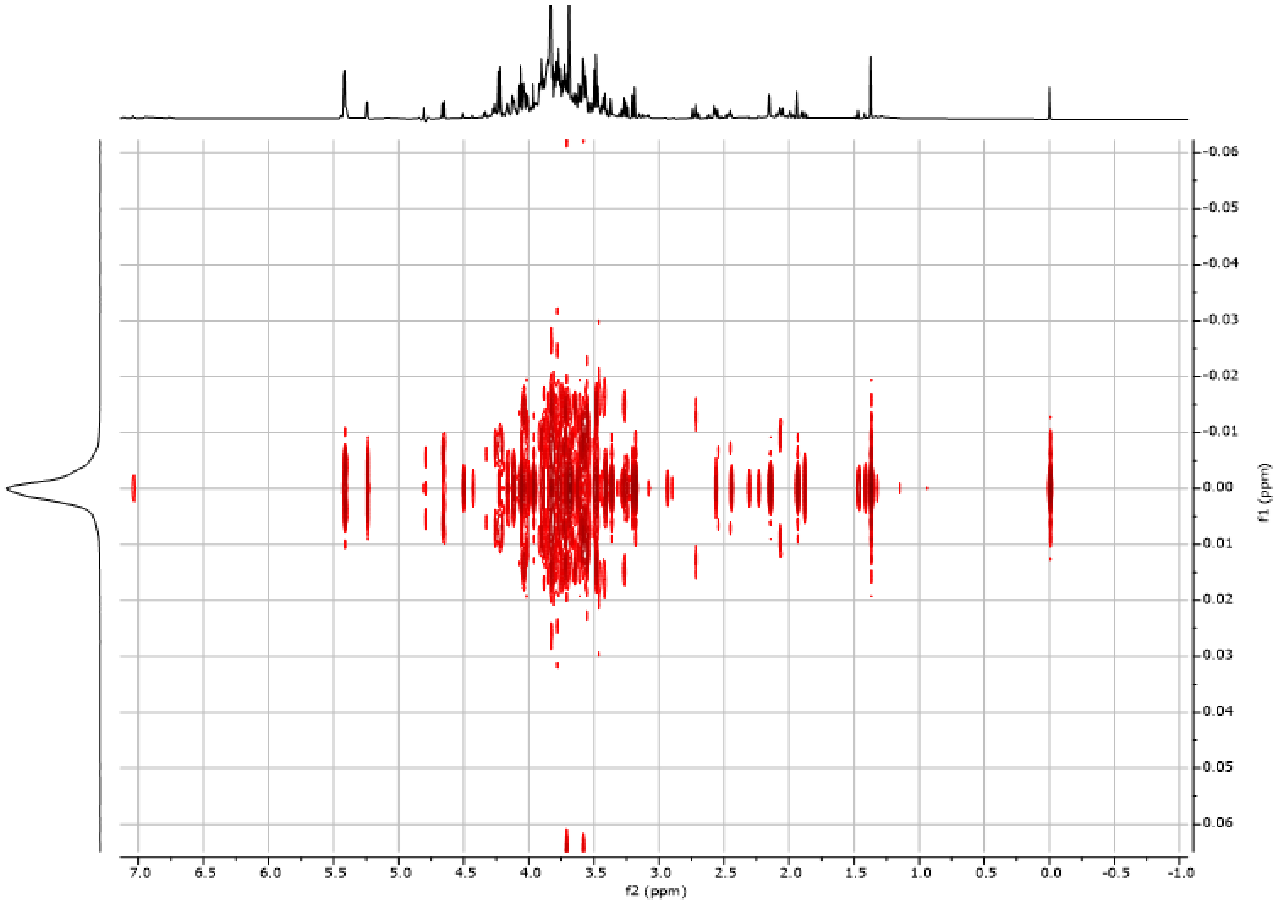

Supplement: Supplementary file 1 [file ijms-27-06757-s001.zip › ijms-4430988-supplementary.pdf]
